# Clinical, immunological and bacteriological characteristics of H7N9 patients nosocomially co-infected by *Acinetobacter Baumannii*: a case control study

**DOI:** 10.1186/s12879-018-3447-4

**Published:** 2018-12-14

**Authors:** William J. Liu, Rongrong Zou, Yongfei Hu, Min Zhao, Chuansong Quan, Shuguang Tan, Kai Luo, Jing Yuan, Haixia Zheng, Jue Liu, Min Liu, Yuhai Bi, Jinghua Yan, Baoli Zhu, Dayan Wang, Guizhen Wu, Lei Liu, Kwok-Yung Yuen, George F. Gao, Yingxia Liu

**Affiliations:** 1 Shenzhen Key Laboratory of Pathogen and Immunity, State Key Discipline of Infectious Disease, Shenzhen Third People’s Hospital, Shenzhen, 518112 China; 2 NHC Key Laboratory of Medical Virology and Viral Diseases, National Institute for Viral Disease Control and Prevention, Chinese Center for Disease Control and Prevention, Beijing, China; 3 CAS Key Laboratory of Pathogenic Microbiology and Immunology, Institute of Microbiology, Chinese Academy of Sciences (CAS), Beijing, China; 4 Department of Epidemiology and Biostatistics, School of Public Health, Peking University, Beijing, China; 5 State Key Laboratory for Emerging Infectious Diseases, The University of Hong Kong, Special Administration Region, Hong Kong, China; 6 Center for Influenza Research and Early-Warning (CASCIRE), Chinese Academy of Sciences, Beijing, China

**Keywords:** Avian influenza A(H7N9) virus, *Acinetobacter baumannii*, Extensively drug-resistant bacteria, Nosocomial infection, Pneumonia, Immune responses

## Abstract

**Background:**

Bacterial co-infection of patients suffering from influenza pneumonia is a key element that increases morbidity and mortality. The occurrence of *Acinetobacter baumannii* co-infection in patients with avian influenza A (H7N9) virus infection has been described as one of the most prevalent bacterial co-infections. However, the clinical and laboratory features of this entity of H7N9 and *A. baumannii* co-infection have not been systematically investigated.

**Methods:**

We collected clinical and laboratory data from laboratory-confirmed H7N9 cases co-infected by *A. baumannii.* H7N9 patients without bacterial co-infection and patients with *A. baumannii*-related pneumonia in the same hospital during the same period were recruited as controls. The antibiotic resistance features and the corresponding genome determinants of *A. baumannii* and the immune responses of the patients were tested through the respiratory and peripheral blood specimens.

**Results:**

Invasive mechanical ventilation was the most significant risk factor for the nosocomial *A. baumannii* co-infection in H7N9 patients. The co-infection resulted in severe clinical manifestation which was associated with the dysregulation of immune responses including deranged T-cell counts, antigen-specific T-cell responses and plasma cytokines. The emergence of genome variations of extensively drug-resistant *A. baumannii* associated with acquired polymyxin resistance contributed to the fatal outcome of a co-infected patient.

**Conclusions:**

The co-infection of H7N9 patients by extensively drug-resistant *A. baumannii* with H7N9 infection is an important issue which deserves attention. The dysfunctions of immune responses were associated with the co-infection and were correlated with the disease severity. These data provide useful reference for the diagnosis and treatment of H7N9 infection.

**Electronic supplementary material:**

The online version of this article (10.1186/s12879-018-3447-4) contains supplementary material, which is available to authorized users.

## Background

Although influenza viruses by itself can cause highly fatal primary influenza pneumonia, the excess mortality rates during influenza pandemics is mainly caused by secondary bacterial pneumonia [1]. Epidemiologic evidence suggests that 95% and 70% of deaths during the influenza pandemic of 1918–19 and 1957–58, respectively, were due to bacterial pneumonia [2]. During the first season (between May 2009 and August 2009) of 2009 pandemic H1N1 influenza, 29% of fatal cases in the United States were associated with a secondary bacterial infection, which was also correlated with the severity of the pneumonia [3]. In hospitalized patients with seasonal influenza, sepsis with or without bacteremia were one of the severe complications, and associated with increased duration of hospitalization days and the requirement of intensive care [4]. The secondary bacterial pneumonia was also observed in patients infected by avian influenza viruses including H5N1 and H7N9 [5]. However, the frequency of secondary bacterial respiratory infection and its effect on the disease severity of avian influenza virus-infected human cases were still lacking.

*Acinetobacter baumannii* is one of the major opportunistic pathogens that have been implicated in various nosocomial infections [6]. The most frequent clinical manifestation of nosocomial *A. baumannii* infection is ventilator-associated pneumonia [7]. Although the clinical impact of nosocomial *A. baumannii* infection has been a matter of continuing debate as many patients with severe influenza had major underlying diseases [8], it has been concluded that *A. baumannii* infection was associated with an increase in attributable mortality, ranging from 7.8 to 23% [9]. *A. baumannii* is attracting much attention owing to the increase in antimicrobial resistance and occurrence of strains that are resistant to virtually all available drugs [10]. The rapid global emergence of multidrug-resistant *A. baumannii* (MDR-AB) strains resistant to all β-lactams, including carbapenems, illustrated the potential of this organism to respond swiftly to changes in selective environmental pressure [11]. Though it is still very rare, recently, resistance to polymyxins has also been described [12], which leads to a pandrug-resistant *A. baumannii* (PDR-AB) that can be fully refractory to the currently available antimicrobial armamentarium.

From March 2013, hundreds of human cases infected by avian influenza A (H7N9) virus with a mortality of 40% were reported and the epidemic situation seems more severe currently with over 100 new cases within December 2016 (http://www.who.int/influenza). Among the H7N9 patients complicated by bacterial infections, MDR-AB was the most common etiology for secondary bacterial pneumonia revealed by independent descriptive studies. Herein, we studied the occurrence of secondary infection by *A. Baumannii* in these severe H7N9 patients. We sought to investigate the role of dysfunctional immunity in the pathogenesis of severe pneumonia in H7N9 patients nosocomially co-infected with *A. baumannii*. Our study may improve the understanding of the high pathogenicity of H7N9 in humans and provide useful recommendations for the clinical diagnosis and treatment.

## Methods

### The patients

From December 2013 to April 2014, 24 patients hospitalized for H7N9 influenza in the Shenzhen Third People’s Hospital, Guangdong, China, which is the designated for H7N9 patients in Shenzhen. The laboratory confirmation of H7N9 virus infection were performed using the protocols as described previously [13, 14]. Briefly, the H7N9 infections of patients were confirmed by real-time RT-PCR assay using influenza subtype-specific primers. Real-time RT-PCR experiments were performed using the RNA which was extracted from the samples using the RNeasy Mini Kit (QIAGEN, Germany). The patients who were diagnosed in other hospitals in Shenzhen were also transferred to our hospital. Meanwhile, we made a surveillance of *A. baumannii* in the hospital. The diagnosis of nosocomial pneumonia by *A. baumannii* was based on the clinical signs of the patients and confirmed by sputum culture and (or) blood culture according to standard microbiological criteria [15]. The patients without typical clinical manifestations were excluded as bacterial colonization of *A. baumannii*. Among the 24 H7N9 patients, 9 patients were diagnosed as secondary infection by *A. baumannii* (H7N9-*A. baumannii* or H7N9-AB case group). Thirteen H7N9 patients without secondary bacterial infection with complete clinical data were termed as H7N9 controls. Fifteen patients with hospital acquired pneumonia due to *A. baumannii*, but H7N9 negative in the same period of our hospital, were also recruited as *A. baumannii* controls. The Declaration of Helsinki was strictly followed. The methods were carried out in accordance with the approved guidelines. We obtained written informed consent from all of the participants or their guardians.

### Clinical data investigation

Data collection (including information prior to and during hospitalization until discharge or death) started immediately after admission and continued on a daily basis. The clinical data were integrated by two independent inputs and checked and verified by the third party. Antimicrobial resistance was determined according to National Committee for Clinical Laboratory Standards guidelines [16].

### The meta-analyses

We screened the previous studies on the clinical descriptions of H7N9 patients published from April 2013 to December 2016, through the US National Library of Medicine National Institutes of Health (www.ncbi.nlm.nih.gov). Eleven publications which described the secondary bacterial infections of H7N9 patients were summarized (Table 1).

**Table 1:** Mutation information in *A. baumannii* SMGC-AB2 isolated from H7N9 patient B4^a^

No.	Gene Annotation	ORF length	position (NT)	mutation (NT)	AA length	position (AA)	substitute (AA)
1	putative permease, YjgP/YjgQ family protein	1101 bp	164	G → A	366	55	Arg → His
2	histidine kinase; PmrB	1335	704	C → T	444	235	Thr → Ile
3	Lipid A phosphoethanolamine transferase, associated with polymyxin resistance, PmrC	1647	1598	A → C	548	533	Lys → Thr
4	NAD-dependent aldehyde dehydrogenase	1575	421	C → T	524	141	Leu → Leu
5	Large repetitive protein, type I secretion C-terminal target domain protein	6657	547	A → G	2218	183	Val → Ile
6	Large repetitive protein, type I secretion C-terminal target domain protein	6657	874	G → A	2218	292	Val → Ile
7	Large repetitive protein, type I secretion C-terminal target domain protein	6657	876	T → C	2218	292	Val → Ile
8	Contig C497	8383 (position)		C → T	Non-coding region		

^a^The reference sequence is *A. baumannii* SMGC-AB1 isolated from H7N9 patient B4 isolated on Day 21 after disease onset. *A. baumannii* SMGC-AB2 was isolated from this patient B4 on Day 26 after disease onset

### Inflammatory mediator tests

We collected the plasma of H7N9-*A. baumannii* co-infected cases and H7N9 controls on the first day of hospitalization and every seven days afterwards. The levels of different cytokines and chemokines were measured with the Bio-Plex ProTM Human Cytokine Array Kits on a Luminex200TM (Luminex®Multiplexing Instrument, Merck Millipore) following the manufacturers’ instructions. The raw data were analyzed using xPONENT 3.1 software (Merck Millipore). Plasmas from seven healthy individuals were used as controls.

### H7N9-specific T cell response tests

The CTL epitope-specific response was measured by performing IFN-γ ELISPOT assays as described previously [17]. Previously identified HLA class I-restricted epitopes (8–11 residues) of influenza virus were retrieved from published data [17, 18]. Conserved peptides between H7N9 and 2009pH1N1 were mixed into a conserved peptide pool as described in reference (JID reference) [19]. Mutated HLA class I-restricted peptides between H7N9 and 2009pH1N1 were considered as H7N9-specific peptide pools (JID reference). The number of spots was determined using an automatic ELISPOT reader and image analysis software (Cellular Technology Limited).

### *A. baumannii* genome sequencing and analyzing

The genomes of the *A. baumannii* isolates SMGC-AB1 and SMGC-AB2 were sequenced by Illumina HiSeq sequencing platform according to manufacturer’s instructions. The 150-bp length pair-end raw reads were first filtered to remove low-quality reads using the DynamicTrim and LengthSort Perl scripts within SolexaQA [20] and then assembled and gap closed using SOAPdenovo2 program (http://soap.genomics.org.cn) [21]. The comparative genome analysis and SNP calling were performed using Mauve software [22]. The draft genomes were annotated using the RAST program (rapid annotation using subsystem technology) [23]. For SNP-based phylogenetic analysis, the whole-genome alignment and SNP calling were performed using Mugsy [24]. The antibiotic resistance gene information was first summarized based on the Subsystem subcategory “Resistance to antibiotics and toxic compounds” according to RAST annotation. Then, the Comprehensive Antibiotic Resistance Database (CARD) [25] was used for the further searching of resistance genes in the sequenced *A. baumannii* genomes.

### Accession codes

The Whole Genome Shotgun projects of patient B4-derived original extensively drug-resistant (XDR) *A. baumannii* SMGC-AB1 and subsequently polymyxin resistant, i.e. a pandrug-resistant isolate (PDR) named *A. baumannii* SMGC-AB2 have been deposited at DDBJ/ENA/GenBank under the accessions NIBI00000000 and NIBH00000000, respectively.

### Statistics

The Student’s *t*-test or χ2-test was used to determine differences between two groups. The Pearson correlation coefficient and Spearman rank correlation coefficient were used for linear correlation analysis. We calculated receiver operating characteristic curves for predictive analysis. Logistic regression was used for multivariate analysis of the risk factors. These statistical analyses were performed with SPSS 16.0 for Windows (SPSS, Inc.). A *P*-value < 0.05 was considered statistically significant.

## Results

### The incidence of *A. baumannii* in H7N9 patients

We have done the meta analyses of the previous studies on the clinical descriptions of H7N9 patients (Additional file 1: Table S1). We found the overall incidence of *A. baumannii* among the reported H7N9 patients was 19.0% (37/195). For the 11 *A. baumannii* cases whose outcome were available, 10 patients (90.9%) was reported fatal, much higher than the crude mortality rate of 32.9% (56/170) amongst H7N9 patients. Most of the identified *A. baumannii* were MDR-AB, and with one case of PDR-AB.

**Fig. 1:**
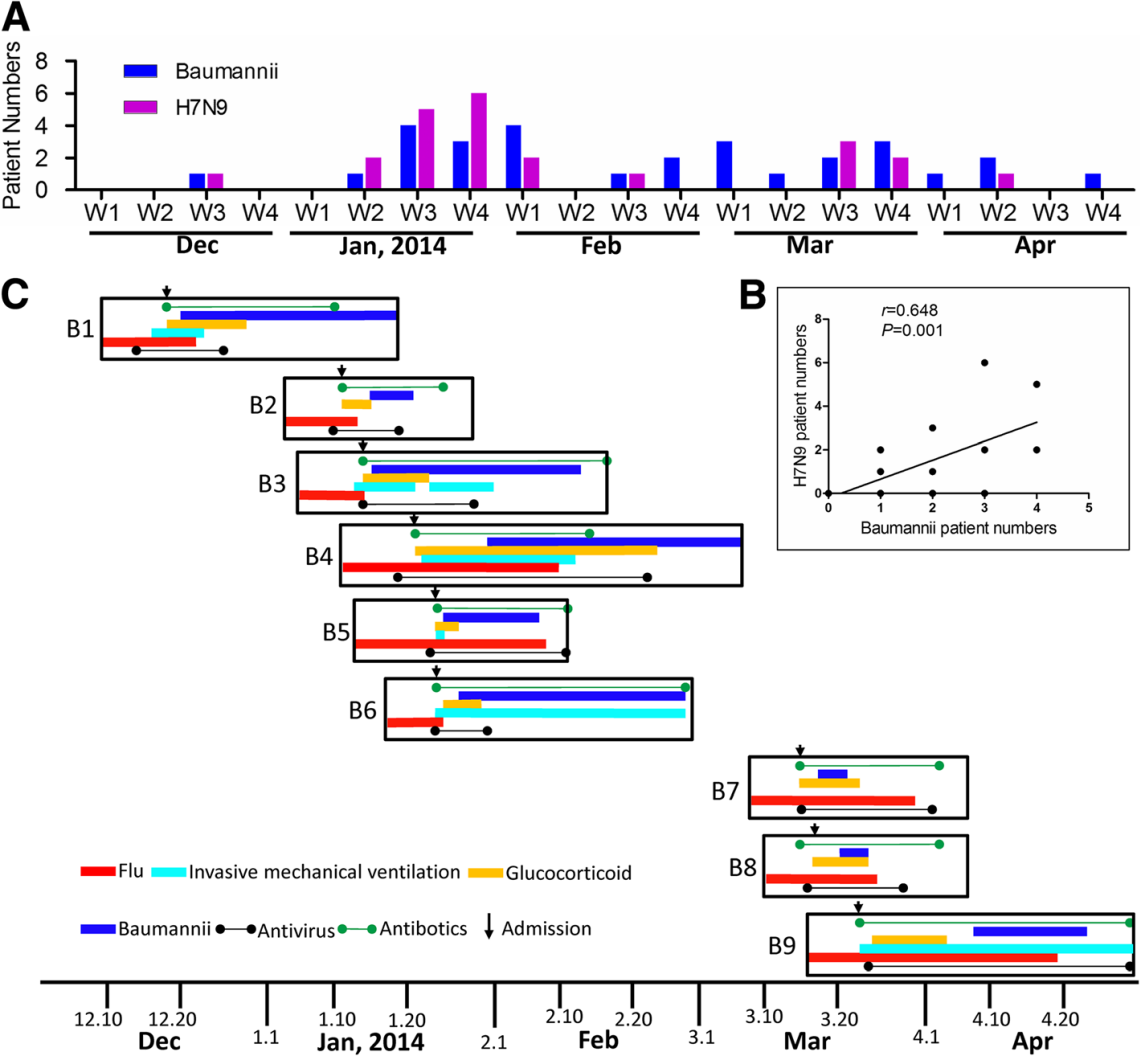
The occurrence of nosocomial *A. baumannii* infection in H7N9 patients during the timeline of the 2013–2014 H7N9 epidemic wave in the hospital. **a**. The weekly reported cases infected by H7N9 and *A. baumannii*, respectively, from December 2013 to April 2014 in Shenzhen Third People’s Hospital, Guangdong China. The first week to the fourth week of each month were denoted as W1 to W4. **b**. The linear correlation of weekly reported numbers of H7N9 patients and *A. baumannii* patients. **c**. The timeline of the laboratory test and clinical treatment of the H7N9 and *A. baumannii* co-infected patients*.* The corresponding dates for timeline were shown below as the horizontal axis. Flu: The persistent period for positive result of H7N9 RNA detected by RT-PCR; Baumannii: The period for *A. baumannii* positive; Admission: the date for the admission of the patient; Antivirus: the period for the anti-influenza drug therapy; Antibiotics: the period for the antibiotic use; Glucocorticoid: the period for glucocorticiod pulse therapy; Invasive mechanical ventilation: the period for the manipulation of invasive mechanical ventilation

From December 2013 to April 2014, 24 patients infected by H7N9 virus were hospitalized in the Shenzhen Third People’s Hospital, Guangdong, China. We found that there were two peaks of hospital admission for H7N9 patients (Fig. 1a, Additional file 2: Table S2 and Additional file 3: Table S3). One is from the late December 2013 to early February 2014 and the second peak is from the early March to early April 2014. Meanwhile, we made a surveillance of positive *A. baumannii* isolation in our hospital and found that the weekly incidence of *A. baumannii* was correlated with the hospitalization of H7N9 patients (Fig. 1b). Among the 24 H7N9 patients, 9 patients (37.5%) were diagnosed as secondary bacterial pneumonia caused by *A. baumannii* (H7N9-*A. baumannii* group) during the hospitalization or on retrospective testing (Fig. 1c). Meanwhile, in the same Infectious Disease Unit which was specially designated for H7N9 admission, none of the other 353 patients without H7N9 were diagnosed as *A. baumannii* infection. This may exclude the possibility that any poor infection control led to a high frequency of this *A. baumannii* infection in H7N9 patients.

### The risk factors for *A. baumannii* co-infection in H7N9 patients

**Fig. 2:**
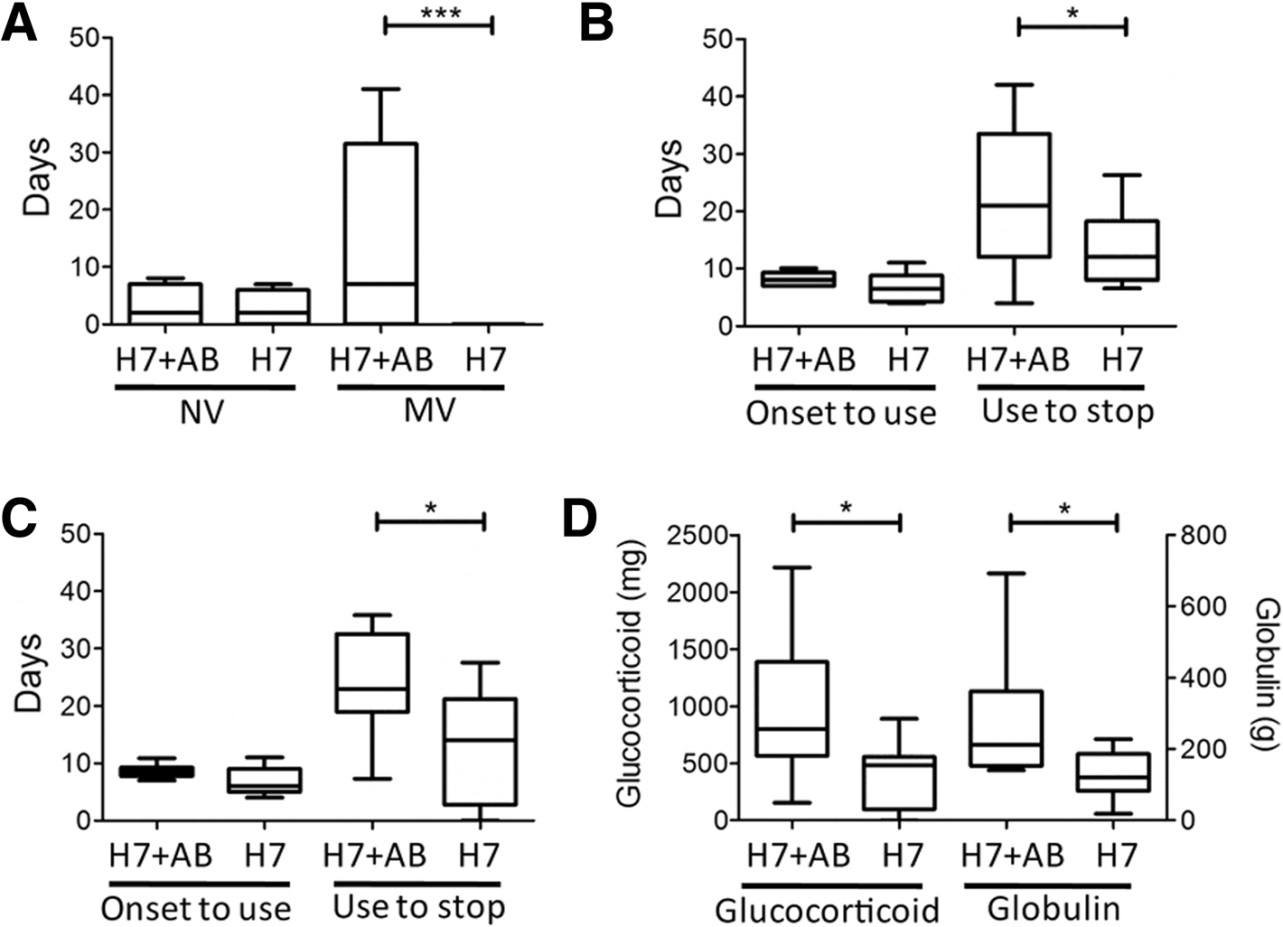
The risk factors for H7N9-*A. baumannii* co-infection. **a**. The comparison of the manipulation of non-invasive ventilation (NV) and invasive mechanical ventilation (MV) between H7N9-*A. baumannii* co-infected cases (H7N9 + AB) and H7N9 control patients without bacterial co-infection (H7N9)*.* **b**. The time from the disease onset to the use of antibiotics (Onset to use) and the total time for the antibiotic use (Use to stop) is compared between H7N9 + AB cases and H7N9 controls. **c**. The time from the disease onset to the use of glucocorticoid (Onset to use) and the total time for the glucocorticoid use (Use to stop) is compared between H7N9 + AB cases and H7N9 controls. **d**. The total dosages for glucocorticoid and gamma globulin compared between between H7N9 + AB cases and H7N9 controls

The median duration from admission to the detection of *A. baumannii* was three days. Based on the time line of the clinical interventions, we found that most of the *A. baumannii* co-infection with H7N9 occurred after the application of invasive mechanical ventilation, antibiotics and broad-spectrum immune intervention drugs such as glucocorticoid (methylprednisolone). Furthermore, the risk factors for the secondary infection of *A. baumannii* in the H7N9 patients were analyzed. We found that the H7N9-*A. baumannii* co-infected patients have a significantly longer duration of invasive mechanical ventilation (Fig. 2a), antibiotics use (Fig. 2b) and glucocorticoid use (Fig. 2c) compared to the 13 H7N9 patients without secondary bacterial infection (H7N9 group, the clinical data for the left two patients are not completed). The total dosages of glucocorticoid and globulin were also higher among H7N9 patients co-infected by *A. baumannii* compared to other H7N9 patients without secondary bacterial infection (Fig. 2d). The multivariate analysis considering the mutual effects of different risk factors showed that invasive mechanical ventilation was still the risk factor for the *A. baumannii* coinfection in H7N9 patients (Additional file 4: Table S4 and Additional file 5: Table S5).

### The clinical impact of *A. baumannii* on H7N9 infection

**Fig. 3:**
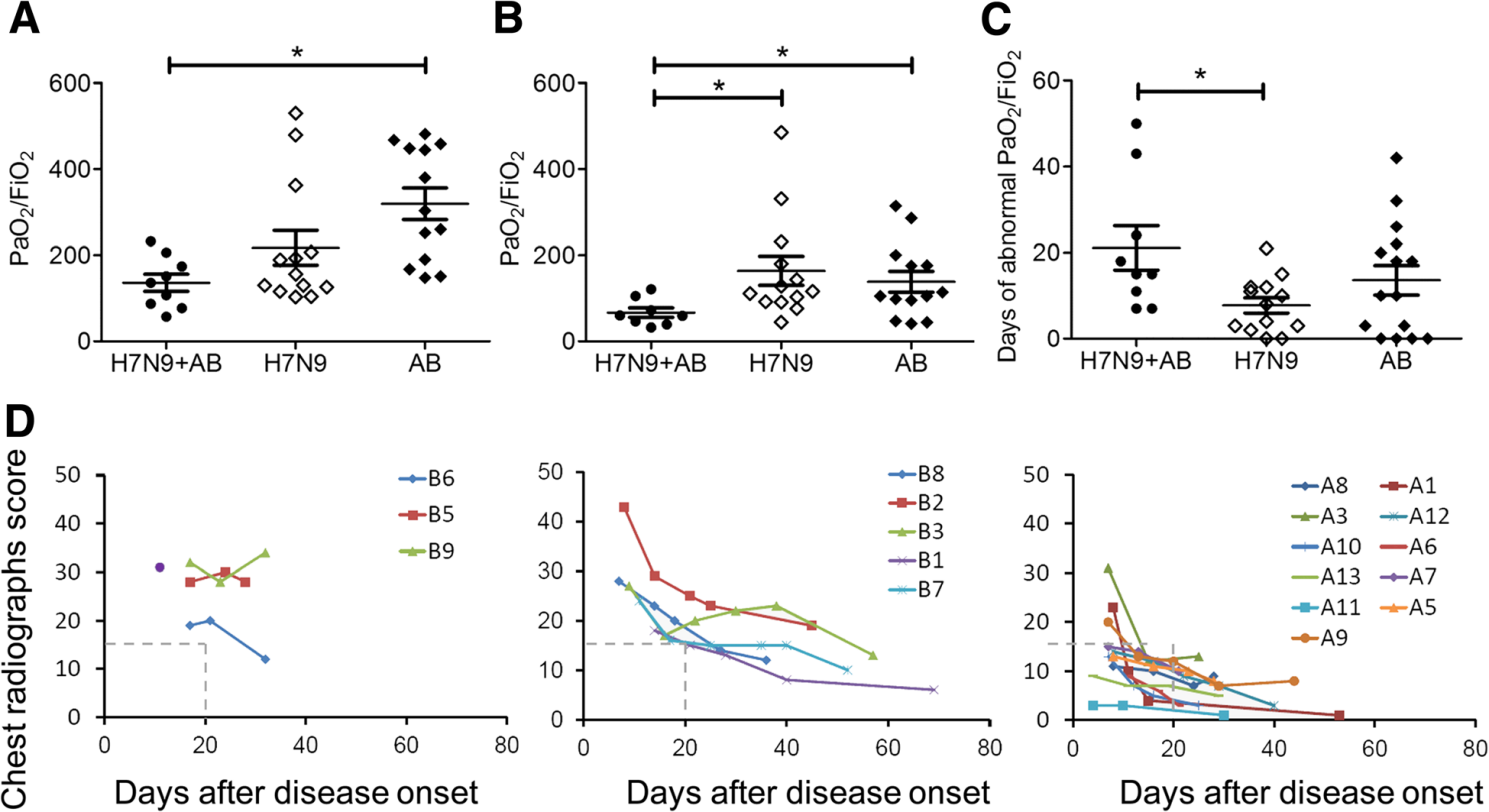
The disease severity of H7N9 patients co-infected by *A. baumannii*. **a**. The oxygenation index (OI, PaO_2_/FiO_2_) on admission compared between H7N9-*A. baumannii* co-infected cases (H7N9 + AB), H7N9 control patients without bacterial co-infection (H7N9), and *A. baumannii* infected control patients with pneumonia (AB group). **b**. The lowest PaO_2_/FiO_2_ of H7N9 + AB cases, H7N9 controls and *A. baumannii* controls during the disease process. **c**. The days for the PaO_2_/FiO_2_ abnormity of the three patient groups. **d**. The longitudinal variation of chest radiographs scores (SRC) during the disease process of H7N9 + AB patients with fatal outcome (Left), survived H7N9 + AB patients (Middle) and H7N9 infection without bacterial co-infection (Right). The SRC = 15 on day 20 was denoted as gray dash lines

The basic demographic characteristics of H7N9 patients co-infected with *A. baumannii* are similar to H7N9 control patients without secondary infection (Additional file 2: Table S2). The average age of H7N9-*A. baumannii* co-infection group was higher than the H7N9 controls but without significance (61.8 ± 18.3 vs 48.2 ± 14.9, *P* > 0.05). No significant differences were found for the clinical manifestations at the time of admission of H7N9-*A.baumannii* cases compared to the H7N9 controls, including the oxygenation index (PaO_2_/FiO_2_) (136.3 ± 60.0 vs 217.3 ± 145.0, P > 0.05) (Additional file 3: Table S3 and Fig. 3a). However, the lowest oxygenation index was lower and the time for the restoring of oxygenation index was longer during the hospitalization process of the H7N9-*A. baumannii* cases than the H7N9 controls (66.8 ± 31.5 vs 163.9 ± 122.1) (Fig. 3b). Furthermore, the recovered times for the abnormal oxygenation indexes in H7N9-*A. baumannii* cases were longer than the H7N9 controls (Fig. 3c), indicating a more severe pulmonary ventilation disorder of the H7N9-*A. baumannii* cases after onset of co-infection by *A. baumannii*. The evaluation of chest radiograph score (SRC) which reflects the lung injury during the infection indicates a prolonged high SRC in the H7N9-*A. baumannii* cases (Fig. 3d). All the SRC of the H7N9-*A. baumannii* cases are still > 15 on day 20 after disease onset, while the SRC of most H7N9 controls without co-infection decreased to < 15 on day 20 after disease onset. The H7N9-*A. baumannii* cases had longer time for fever duration (14.3 ± 9.2 vs 8.1 ± 2.5) and the length of hospital are also longer than the H7N9 controls without co-infection (30.0 ± 11.2 vs 15.5 ± 7.1). All H7N9 controls without co-infection survived, but four of the nine (44.4%) H7N9-*A. baumannii* patients died. In the H7N9-*A. baumannii* cases, the patients who died had persistent abnormal oxygenation index compared to that of a gradual improvement in the survivors (Additional file 6: Figure S1).

### Dysfunctional immunity in co-infected patients

**Fig. 4:**
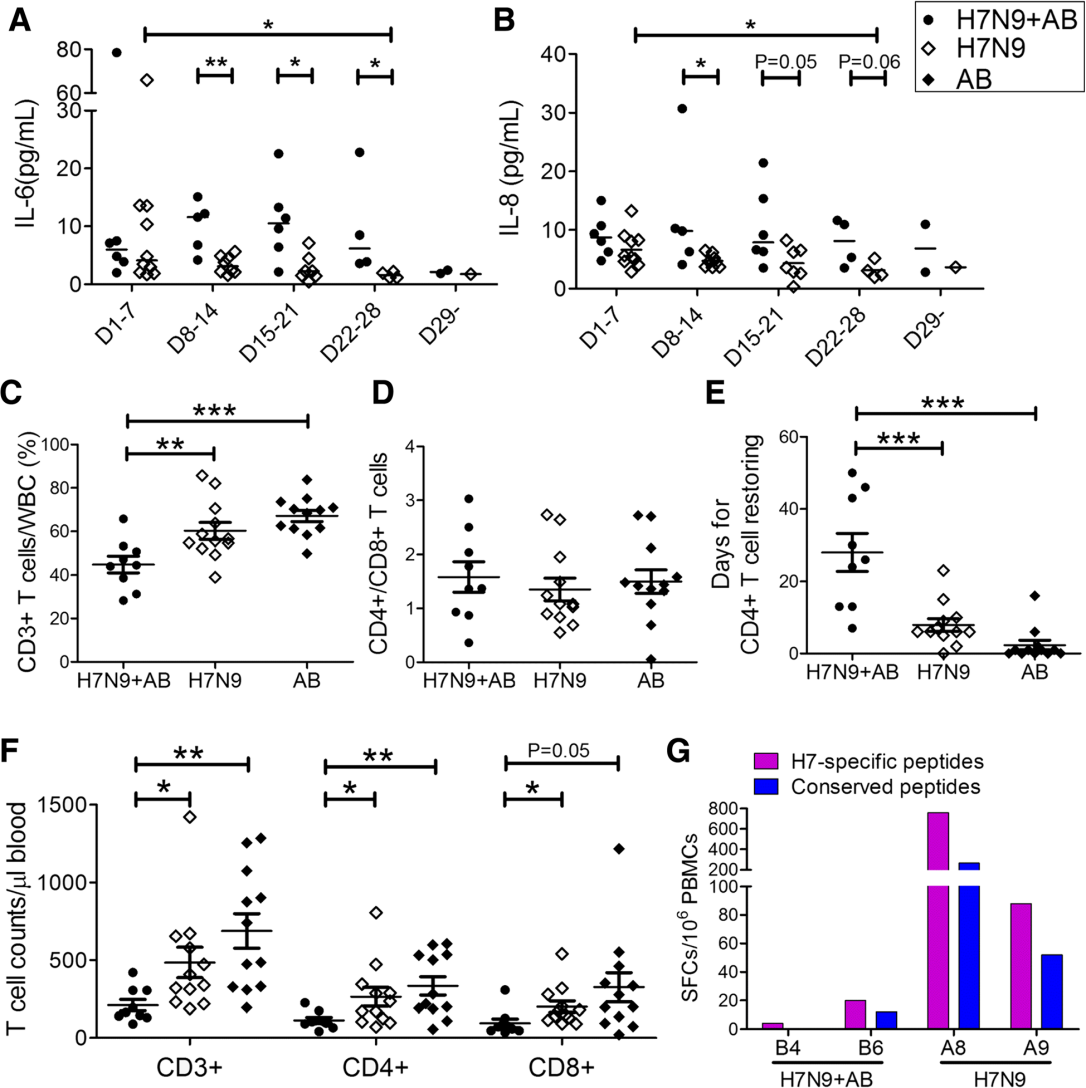
Dysfunctional immunity in H7N9 patients co-infected by *A. baumannii.* The plasma cytokines IL-6 (**a**) and IL-8 (**b**) in the H7N9*-A. baumannii* co-infected patients (H7N9 + AB) are higher than the H7N9 control patients (H7N9 group) during week 2 (Day 8–14), week 3 (Day 15–21) and week 4 (Day 22–28) after the admission. In contrast, IL-6 and IL-8 in the H7N9 control patients were decreasing gradually from week 1 (Day 1–7) to week 4 (Day 22–28) after hospitalization. Through the analysis of T-lymphocyte subtypes, the ratio of T-cells in total white blood cells on admission (**c**) was compared between H7N9 + AB cases and H7N9 controls and *A. baumannii* pneumonia control patients (AB group). The ratio of CD4^+^ T-cells/CD8^+^ T-cells (**d**) was also calculated among cases and controls. The time for CD4^+^ T-cell count restoring was longer for H7N9 + AB patients (**e**). The CD3^+^, CD4^+^ and CD8^+^ T-cell counts were compared between the co-infected cases and controls (**f**). The influenza virus-specific T-cell responses (**g**) were detected through ELISPOT by using the freshly-isolated PBMCs from the patients. H7N9-specific peptide pools and conserved peptide pools were used as the stimulators

IL-6 and IL-8 are two major representative cytokines which can reflect the disease severity during the acute phase of H7N9 as reported previously [26]. Furthermore, the cytokines IL-6 and IL-8 among H7N9 patients without *A. baumannii* presented a decreasing trend during the disease course (Fig. 4a and b). In contrast, both the IL-6 and IL-8 among the plasma of the H7N9-*A. baumannii* patients increased in the third week after disease onset and remained higher than the H7N9 patients until the fifth week after disease onset or death.

The T-cell counts correlated with the lung function of the H7N9 patients (Additional file 7: Figure S2). We next investigated the T-cell subgroups of the H7N9-*A. baumannii* cases. We found that the patients who had co-infection by *A. baumannii* have fewer CD4^+^ and CD8^+^ T-cells, compared to H7N9 controls without co-infection, and also lower than the *A. baumannii* controls without H7N9 infection (Fig. 4c-f). Generally, the normalization time for the CD4^+^ T-cell count among the H7N9-*A. baumannii* cases are also longer than those of the H7N9 controls and *A. baumannii* controls (Fig. 4e). For the longitudinal profile of the lymphocyte count, fatal patients with H7N9-*A. baumannii* co-infection had a consistently low level of both CD4^+^ and CD8^+^ T-cell counts, while the survived H7N9-*A. baumannii* patients have gradual normalization of T-cell counts but still much slower than the H7N9 controls without co-infection (Additional file 8: Figure S3).

We performed biostatistical analyses to further identify correlations between immune factors as of H7N9-*A. baumannii* co-infection. We first used SPSS software to calculate its predictive value for co-infection. The area under the curve (AUC) of plasma IL-6 and IL-8 levels were 0.927 and 0.850 respectively during the second week of hospitalization among H7N9-infected patients, and were 0.881 and 0.833 during the third week (Additional file 9: Figure S4). In addition, the CD3^+^ T-cell counts (AUC: 0.846), CD8^+^ T-cell counts (AUC: 0.812) on admission and the restoring time for the abnormal CD4^+^ T-cell counts (AUC: 0.876) were all predictive for the H7N9-*A. baumannii* co-infection. Additionally, the traditional biomarkers of bacterial infection, procalcitonin and C-reactive protein levels in blood fluctuated during the disease process, but the two indicators of fatal cases with H7N9-*A. baumannii* co-infection remained at a higher level during a longer period compared to survived cases (Additional file 10: Figure S5 and Additional file 11: Figure S6). We also used multivariate analysis in logistic regression to further analyze the independent predictor for *A. baumannii* co-infection among patients with H7N9 infections (Additional file 4: Table S4 and Additional file 5: Table S5). CD4^+^ T-cell counts in blood harvested on the day of admission were found to be an independent indicator among the clinical parameters of H7N9-infected patients including procalcitonin and C-reactive protein.

### Lower virus-specific T-cell responses amongst H7N9-*A. baumannii* co-infected patients

The influenza virus-specific T-cell tests through the ELIPOST assays by the stimulation of H7N9-derived peptides or conserved peptides, indicated that H7N9-*A. baumannii* patients had a weak CD8^*+*^ T-cell responses to H7N9 virus compared to H7N9 controls without co-infection (Fig. 4g).

### The antibiotic resistance features of the *A. baumannii* noscomially acquired by H7N9 patients

After an average time of 6 days of invasive ventilation, the H7N9 patients were detected to be *A. baumannii* positive. According to standard drug resistance definition [27], the original isolates from six patients were identified as extensively drug-resistant (XDR). As the *A. baumannii* strains were susceptible to polymyxin, the patients were then treated with polymyxin B with an average total dosage of 14.7 million units for an average 10.5 days. Notably, for patient B4, after polymyxin treatment for five days, the original XDR isolate SMGC-AB1 became polymyxin resistant, i.e. a pandrug-resistant isolate (PDR) named SMGC-AB2 (Table 1, Additional file 12: Table S6).

### Rapid emergence of a pandrug-resistant isolate featured by candidate polymyxin resistance-associated mutations

To gain insights into the genetic features of the H7N9 patient infected with *A. baumannii*, the whole genomes of both SMGC-AB1 and SMGC-AB2 were sequenced. SNP-based phylogenic analysis showed that SMGC-AB1 was clustered into the European clone (EC) II group, including MDR-ZJ06, MDR-TJ, BJAB0868 and BJAB07104 from Mainland China, TYTH-1 and TCDC-AB0715 from China Taiwan and AB1656–2 from Korean (Additional file 13: Figure S7). The SMGC-AB1 genome harbored a total of 57 genes belonging to Subsystem subcategory “Resistance to antibiotics and toxic compounds” according to RAST annotation (Additional file 14: Figure S8). Notably, SMGC-AB1 encodes 6 β-lactamases and 2 aminoglycoside adenylyl-transferases.

When comparing the genome of SMGC-AB1 with its polymyxin resistance descendant, SMGC-AB2, only 8 nucleotide point mutations were identified in SMGC-AB2, 7 of which were located in coding regions and 6 lead to nonsynonymous mutation (Table 1). The 1st mutation was located in a putative permease protein encoding gene belonging to YjgP/YjgQ family (now LptF/LptG), which has been identified to be a component of ABC transporter for lipopolysaccharide transport to the cell surface [28]. Notably, the 2nd and 3rd mutations were located in the *pmrCAB* operon, a gene cluster encoding the PmrC phosphoethanolamine phosphotransferase involved in polymyxin resistance [29]; mutations in genes of *pmrB* and *pmrC* leading to Thr → Ile (T235I) and Lys → Thr (L533 T) substitution, respectively. The 5th to 7th mutations causing Val → Ile (V183I and V292I) are all located in a large repetitive protein harboring type I secretion C-terminal target domain. The 4th mutation resulting in no amino acid substitution appeared in a gene encoding NAD-dependent aldehyde dehydrogenase, and the 8th lies in the non-coding region.

## Discussion

It is known that secondary bacterial pneumonia during influenza virus infection is a major cause for high mortality of severe or fatal cases. Compared with the dominant bacterial infection of *Streptococcus pneumoniae* and *Staphylococcus aureus* in other influenza virus pandemics [30], *A. baumannii* is the most commonly encountered pathogen in sputum or endotracheal samples in the H7N9 virus infected clinical cases [31]. Here, we presented a typical cohort of the H7N9 patients confected by *A. baumannii* and elucidated their dysfunctional immune responses. Furthermore, the genome features of the *A. baumannii* and particularly the genome variations contributed to the acquired polymyxin resistance which might have led to the fatal outcome of this patient.

The role of the hospital environment as a reservoir for *A. baumannii* has been well defined [32]. Hospital equipment, especially the medical equipment for invasive mechanical ventilation, is one of the important risk factors that predispose individuals to the acquisition of, and infection with, *A. baumannii* [6]. Other risk factors include prior antibiotic use and prior use of broad-spectrum drugs [33]. In our study, H7N9 patients co-infected by *A. baumannii* have a longer use of antibiotics, glucocorticoid, and the invasive mechanical ventilation, all of which may predispose to the occurrence of the *A. baumannii* co-infection in these patients.

In fact, influenza virus infection itself also enhances the susceptibility of the patients to secondary bacterial infection by its impacts on the inflammatory signals and function of early innate immune defense [34]. It is known that levels of inflammatory mediators such as IL-6 and IL-18 were higher in the patients with co-infection of influenza virus and bacteria than in patients with bacterial pneumonia or influenza virus infection alone [35]. Concordantly, in our study, the IL-6 and IL-8 increased to a higher level in the H7N9-*A. baumannii* co-infected patients compared to H7N9 patients without bacterial infection.

Influenza A infection could also increase susceptibility to secondary bacterial pneumonia by dysregulation of different innate immune cells. The influenza virus can inhibit Th17 immunity by the induction of type I IFNs [36] and IL-27 [37], and also can inhibit neutrophil attraction through the mediation of protein Setdb2 [38] and the production of IL-10 [39]. In our study, we found that the H7N9 patients with a persistently low level of CD8^+^ and CD4^+^ T-cell population, especially hyporesponsive antigen-specific T-cell responses, are susceptible to the *A. baumannii* infection and the subsequent fatal outcome. This may indicate a T-cell anergy after the avian H7N9 influenza virus infection as previously defined in severe H1N1 infected patients [40].

It is surprising that SMGC-AB1 quickly acquired resistance to polymyxin, an antibiotic that is considered to be the last hope to cope with MDR and or XDR gram-negative bacteria. Polymyxins are cyclic cationic peptides with a long hydrophobic tail that interact with the lipid A moiety of lipopolysaccharide (LPS) to disrupt the integrity of outer membranes of gram-negative bacteria. Three major resistance mechanisms to polymyxin in bacteria have been identified: modification of the bacterial outer membrane lipopolysaccharide, proteolytic cleavage of the drug and activation of broad-spectrum efflux pumps, among which modification of lipid A of LPS regulated by the two-component regulatory system PmrAB has been frequently reported in *A. baumannii* [41]. It is reported that a single mutation in *pmrB* gene causing T235I amino acid substitution can lead to 16-fold increases in polymyxin B MIC in *A. baumannii* [29]. In this study, we found that the SMGC-AB2 harbors a mutation in *pmrB* gene exactly resulting in T235I amino acid substitution; we believe that this mutation plays a decisive role for this isolate to resist polymyxin. As demonstrated, mutations in the *pmrA* or *pmrB* genes usually result in the constitutive expression of *pmrC*, thus leading to LPS modification and reduction of the affinity of polymyxins [42]. In clinical resistant isolates, similar mutations have also been found in *pmrC* gene.^.^ However, in SMGC-AB2, the mutation located at the end of the C-terminus of pmrC protein (L533 T), beyond the functional sulfatase domain (aa 237–532), may not contribute to the resistance. It is reported that *A. baumannii* can also develop resistance to polymyxin by mutating genes responsible for LPS production [43]. Though no mutations were found in the biosynthesis pathway of LPS in SMGC-AB2, a mutation appeared in a pupative permease (Table 1, mutation No. 1) that probably functions in LPS transport. We speculate that this mutation may cause the decrease of LPS production, thus leading to the decrease of the drug target, which may also play a secondary role in the resistance phenotype of SMGC-AB2. Finally, we also found three mutations in a large repetitive protein harboring type I secretion C-terminal target domain leading to two amino acid substitutions (Val → Ile); whether and how this protein and the mutations are involved in the polymyxin resistance in SMGC-AB2 needs further investigations.

In conclusion, we described the occurrence of secondary infection of *A. baumannii* and its impacts on the disease severity in H7N9 patients. The dysfunctional immunity in the H7N9 patients correlated to the *A. baumannii* co-infection. Furthermore, the genome variations of *A. baumannii* contributed to the acquired polymyxin resistance in the patient with fatal outcome. We suggest that the enhancement of the anti-nosocomial infection measures for the prevention of *A. baumannii* in the H7N9 patients with risk factors for secondary infections, and the early administration of appropriate antibiotic regimen when such co-infection is detected by frequent microbiological testing.

## Conclusions

Invasive mechanical ventilation is the most significant risk factor for the nosocomial *A. baumannii* co-infection in in patients with avian influenza A (H7N9) virus infection. The occurrence of H7N9 co-infection with *A. baumannii* is a key factor for the severity of the patients, with a manifestation of lower oxygenation indexes and chest radiograph scores. Dysregulation of immune responses in the H7N9 patients correlates to the susceptibility of *A. baumannii* co-infection and severe clinical manifestation. Novel polymyxin resistance-associated genome variations of *A. baumannii* could quickly emerge during the disease process and may contribute to fatal outcome of the H7N9-*A. baumannii* co-infected patients. Both the immune and bacterial features in the patients may contribute to and act as biomarkers for the severe pneumonia and fatal outcome of the co-infection. These conclusions shed light on the pathomechanism, diagnosis and treatment of H7N9 and bacterial co-infections.

## Additional files

Additional file 1: **Table S1.** The meta analyses of *A. baumannii* infection in H7N9 patients. (DOCX 59 kb)

Additional file 2: **Table S2.** Basic characters of the patients. (DOCX 34 kb)

Additional file 3: **Table S3.** Clinical presentation and main lab-findings on admission. (DOCX 34 kb)

Additional file 4: **Table S4.** The multivariate analysis of the risk factors of H7N9 patients co-infected by A. baumannii (*n* = 13) compared to H7N9 control patients (*n* = 9). (DOCX 32 kb)

Additional file 5: **Table S5.** The multivariate analysis of the clinical risk factors and indicators of H7N9 patients co-infected by *A. baumannii* (*n* = 13) compared to the group (*n* = 24) combined of H7N9 control and *A. baumannii* control patients. (DOCX 32 kb)

Additional file 6: **Figure S1.** The longitudinal variation of oxygenation index (PaO_2_/FiO_2_) in the H7N9 patients co-infected by *A. baumannii*. (PDF 190 kb)

Additional file 7: **Figure S2.** The association of T-cell counts with oxygenation index (PaO_2_/FiO_2_) in the H7N9 patients. (PDF 237 kb)

Additional file 8: **Figure S3.** The longitudinal trends of CD3^+^ T-cell counts, CD4^+^ T-cell counts and CD8^+^ T-cell counts among the H7N9 patients. (PDF 210 kb)

Additional file 9: **Figure S4.** ROC curve of the plasma levels of cytokines and T-cell characteristics in H7N9 patients. (PDF 431 kb)

Additional file 10: **Figure S5.** The longitudinal variation of PCT in the H7N9 patients co-infected by *A. baumannii*. (PDF 185 kb)

Additional file 11: **Figure S6.** The longitudinal variation of CRP in the H7N9 patients co-infected by *A. baumannii*. (PDF 188 kb)

Additional file 12: **Table S6.** Antibiotic susceptibility profiles for *A. baumannii* SMGC-AB1 and SMGC-AB2. (DOCX 32 kb)

Additional file 13: **Figure S7.** Phylogenetic position of *A. baumannii* SMGC-AB1 and its polysaccharide antigen gene clusters. (PDF 624 kb)

Additional file 14: **Figure S8.** Comparison of gene distribution in Subsystem subcategory “Resistance to antibiotics and toxic compounds”. (PDF 337 kb)
