# Supplementary material for: Clinical, immunological and bacteriological characteristics of H7N9 patients nosocomially co-infected by Acinetobacter Baumannii: a case control study
Source: BMC Infect Dis. 2018 Dec 14;18:664. doi: 10.1186/s12879-018-3447-4 (PMC6295110; doi:10.1186/s12879-018-3447-4)
Supplement: Supplementary file 1 — Table S1. The meta analyses of A. baumannii infection in H7N9 patients. (DOCX 59 kb) [file 12879_2018_3447_MOESM1_ESM.docx]

**Table S1. The meta analyses of *A. baumannii* infection in H7N9 patients ^a^.**

| **Reference** |  | **Patient number** | **Baumannii infection** | **Drug-resistance** | **Specimen** |
| --- | --- | --- | --- | --- | --- |
| **(Rongbao Gao, NEJM 2013)[**[**1**](#_ENREF_1)**]** |  | 3(3)^b^ | 2(2) | Carbapenem-resistant | Lower respiratory tract specimens |
| **(Hai-Nv Gao, NEJM 2013)[**[**2**](#_ENREF_2)**]** |  | 111(30) | 17(- ^c^) | - | Sputum/endotracheal samples |
| **(Xiao Tang, Clin Respir J, 2014)[**[**3**](#_ENREF_3)**]** |  | 1(1) | 1(1) | Pandrug-resistant | Sputum |
| **(Liang Yu, CID, 2013)[**[**4**](#_ENREF_4)**]** |  | 12(6) | 4(3) | Multidrug-resistant | Sputum |
| **(Yan-rong Wang, Int J Infect Dis,2014)[**[**5**](#_ENREF_5)**]** |  | 1(0) | 1(0) | Multidrug-resistant | Sputum and blood |
| - **(**[**Y. Hu**](http://www.sciencedirect.com.proxy2.library.illinois.edu/science/article/pii/S1198743X15003705)**,** [**Clin Microbio Infect**](http://www.sciencedirect.com.proxy2.library.illinois.edu/science/journal/1198743X)**, 2015)[**[**6**](#_ENREF_6)**]** |  | 1(1) | 1(1) | - | Blood |
| **(Shuihua Lu, PLoS ONE, 2014)[**[**7**](#_ENREF_7)**]** |  | 18(6) | 3(-) | - | Blood/tracheal aspiration |
| **(Yang Pan,J Infect Dev Ctries,2015)[**[**8**](#_ENREF_8)**]** |  | 1(1) | 1(1) | Multidrug-resistant | Blood |
| **(Li Guo, Emerg Infect Dis, 2014)[**[**9**](#_ENREF_9)**]** |  | 21(7) | 1(1) | - | - |
| **(Haifeng Lu, BMC Infect Dis,2014)[**[**10**](#_ENREF_10)**]** |  | 25(-) | 5(-) | Multidrug-resistant | Sputum |
| **(Guyi Wang, Southeast Asian J Tropic Med Pub Heal,2015)[**[**11**](#_ENREF_11)**]** |  | 1(1) | 1(1) | Multidrug-resistant | Sputum and blood |
| **Total** |  | 170(56, 33%) ^d^, | 11(10, 91%) ^d^ |  |  |

^a^ The literatures from April 2013 to December 2016 were summarized.

^b^ The number in bracket denotes the fatal cases.

^c^ The data not available is shown as hyphen "-".

^d^ The literatures with confirmed numbers of the patients and the fatal cases were involved for the calculation of total number. The mortality rates are shown as percentages.

**References**

1. Gao R, Cao B, Hu Y, Feng Z, Wang D, Hu W, Chen J, Jie Z, Qiu H, Xu K *et al*: **Human infection with a novel avian-origin influenza A (H7N9) virus**. *N Engl J Med* 2013, **368**(20):1888-1897.

2. Gao HN, Lu HZ, Cao B, Du B, Shang H, Gan JH, Lu SH, Yang YD, Fang Q, Shen YZ *et al*: **Clinical findings in 111 cases of influenza A (H7N9) virus infection**. *N Engl J Med* 2013, **368**(24):2277-2285.

3. Tang X, He H, Sun B, Wan J, Ban C, Zhang C, Wang S, Xia J, Li J, Liu Y *et al*: **ARDS associated with pneumonia caused by avian influenza A H7N9 virus treated with extracorporeal membrane oxygenation**. *Clin Respir J* 2015, **9**(3):380-384.

4. Yu L, Wang Z, Chen Y, Ding W, Jia H, Chan JF, To KK, Chen H, Yang Y, Liang W *et al*: **Clinical, virological, and histopathological manifestations of fatal human infections by avian influenza A(H7N9) virus**. *Clin Infect Dis* 2013, **57**(10):1449-1457.

5. Wang YR, Li JM, Wang XF: **Clinical and epidemiological analysis of the first case of human infection with avian influenza A (H7N9) virus in Shenzhen, China**. *InternJ Infect Dis* 2014, **25**:177-179.

6. Hu Y, Ren X, Liu Y, Yang F, Liu H, Cao B, Jin Q: **Serial high-resolution analysis of blood virome and host cytokines expression profile of a patient with fatal H7N9 infection by massively parallel RNA sequencing**. *Clin Microbiol Infect* 2015, **21**(7):713 e711-714.

7. Lu S, Li T, Xi X, Chen Q, Liu X, Zhang B, Ou J, Liu J, Wang Q, Zhu B *et al*: **Prognosis of 18 H7N9 avian influenza patients in Shanghai**. *PloS ONE* 2014, **9**(4):e88728.

8. Pan Y, Shi W, Yang P, Wu S, Peng X, Cui S, Zhang D, Lu G, Zhao J, Liu Y *et al*: **A case of human infection with avian Influenza A/H7N9 virus in Beijing: virological and serological analysis**. *J Infect Devel Count* 2015, **9**(3):317-320.

9. Huang F, Guo J, Zou Z, Liu J, Cao B, Zhang S, Li H, Wang W, Sheng M, Liu S *et al*: **Angiotensin II plasma levels are linked to disease severity and predict fatal outcomes in H7N9-infected patients**. *Nat Commun* 2014, **5**:3595.

10. Lu H, Zhang C, Qian G, Hu X, Zhang H, Chen C, Liang W, Gao H, Yang Y, Li L: **An analysis of microbiota-targeted therapies in patients with avian influenza virus subtype H7N9 infection**. *BMC Infect Dis* 2014, **14**:359.

11. Wang G, Zhou Y, Gong S, Dong H, Wu G, Xiang X, Tang J: **A pregnant woman with avian influenza A (H7N9) virus pneumonia and ARDS managed with extracorporeal membrane oxygenation**. *Southeast Asian J Trop Med Pub Heal* 2015, **46**(3):444-448.
