# Supplementary material for: Clinical, immunological and bacteriological characteristics of H7N9 patients nosocomially co-infected by Acinetobacter Baumannii: a case control study
Source: BMC Infect Dis. 2018 Dec 14;18:664. doi: 10.1186/s12879-018-3447-4 (PMC6295110; doi:10.1186/s12879-018-3447-4)
Supplement: Supplementary file 2 — Table S2. Basic characters of the patients. (DOCX 34 kb) [file 12879_2018_3447_MOESM2_ESM.docx]

**Table S2. Basic characters of the patients.**

| **Characteristics** | **H7N9^a^** | **H7N9+*A. baumannii*^b^** | ***A. baumannii*^c^** | ***Ｐ*^d^ (A&B)** | ***Ｐ*^e^ (B&C)** | **Case B4^f^** |
| --- | --- | --- | --- | --- | --- | --- |
| Case number | 13 | 9 | 15 |  |  | 1 |
| Age (yr, mean±SD)^g^ | 48.2±14.9 | 61.8±18.3 | 49.5±21.0 | 0.07 | 0.13 | 34 |
| Sex (M) , n (%) | 7(53.8)^d^ | 6(66.7) | 11(73.3) | 0.87 | 0.91 | F |
| Comorbidities , n (%) |  |  |  |  |  |  |
| Malignancy | 0 | 0 | 0 | NA | NA | 0 |
| COPD | 0 | 1(11.1) | 5(33.3) | 0.85 | 0.47 | 0 |
| Asthma | 0 | 0 | 0 | NA | NA | 0 |
| Bronchiectasis | 0 | 0 | 1(6.7) | NA | 0.79 | 0 |
| Diabetes | 2(15.4) | 3(33.3) | 2(13.3) | 0.64 | 0.52 | 0 |
| Current smoker, n (%) | 3(23.1) | 3(33.3) | 4(26.7) | 0.96 | 0.91 | 0 |
| Previous antibiotic, n (%) | 13(100) | 9(100) | 15(100) | NA | NA | 1 |
| Poultry contacts, n (%) | 9(69.2) | 6(66.7) | 0 | 0.73 | 0.002 | 1 |

^a^ H7N9 patients without bacterial co-infection in the lung are shown here as the H7N9 controls.

^b^ H7N9+*A. baumannii*: H7N9 patients coinfected by Acinetobacter baumannii in the lung.

^c^ *A. baumannii*: Patients with *Acinetobacter baumannii* related pneumonia are shown here as *A. baumannii* controls.

^d^ The P value between H7N9 group and H7N9+ *A. baumannii.* The data presented as the number (percentage) of patients were analyzed by Chi-square tests, otherwise were analyzed by t test.

^e^ The P value between H7N9+*A. baumannii* group and *A. baumannii* group. The data presented as the number (percentage) of patients were analyzed by Chi-square tests, otherwise were analyzed by t test.

^f^ The case B4 was the patient whose *A. baumannii* genomes were sequenced.

^g^ Data are presented as the number (percentage) of patients unless indicated otherwise.
