# Supplementary material for: Clinical, immunological and bacteriological characteristics of H7N9 patients nosocomially co-infected by Acinetobacter Baumannii: a case control study
Source: BMC Infect Dis. 2018 Dec 14;18:664. doi: 10.1186/s12879-018-3447-4 (PMC6295110; doi:10.1186/s12879-018-3447-4)
Supplement: Supplementary file 3 — Table S3. Clinical presentation and main lab-findings on admission. (DOCX 34 kb) [file 12879_2018_3447_MOESM3_ESM.docx]

**Table S3. Clinical presentation and main lab-findings on admission.**

| **Characteristics** | **H7N9^a^** | **H7N9+*A. baumannii*^b^** | ***A. baumannii*^c^** | ***Ｐ* (A&B)^d^** | ***Ｐ* (B&C)^e^** | **Case B4^f^** |
| --- | --- | --- | --- | --- | --- | --- |
| Case number | 13 | 9 | 15 |  |  | 1 |
| **Main symptoms** |  |  |  |  |  |  |
| Cough, n (%)^g^ | 13(100) | 9(100) | 11(73.3) | NA | 0.26 | 1 |
| Sputum, n (%) | 8(61.5) | 7(77.8) | 10(33.3) | 0.74 | 0.91 | 1 |
| Hemoptysis, n (%) | 1(7.7) | 0 | 1(6.7) | 0.85 | 0.79 | 0 |
| Sore throat, n (%) | 0 | 1(11.1) | 1(6.7) | 0.85 | 0.70 | 0 |
| Temperature, °C(mean ± SD) | 39.3±0.6 | 39.5±0.8 | 38.5±1.1 | 0.48 | **0.017** | 39.5 |
| Fever duration, days(mean ± SD) | 8.1±2.5 | 14.3±9.2 | 10.8±11.2 | **0.030** | 0.49 | 11 |
| **Main lab-findings on admission** |  |  |  |  |  |  |
| Hemoglobin, g/L | 133.8±13.9 | 132.6±13.7 | 119.3±20.9 | 0.86 | 0.18 | 126 |
| leukocyte, ×10^9^cells/L | 4.4±1.3 | 5.1±2.3 | 13.5±6.8 | 0.35 | **0.0025** | 1.2 |
| Neutrophils, % | 78.7±8.3 | 81.5±8.4 | 71.4±25.1 | 0.44 | 0.26 | 77.5 |
| Lymphocytes, % | 16.3±5.5 | 13.6±8.8 | 11.2±4.9 | 0.39 | 0.43 | 18.3 |
| Platelets, ×10^9^cells/L | 129.8±38.6 | 164.7±45.4 | 232.4±133.4 | 0.086 | 0.15 | 128 |
| Albumin, g/L | 36.9±2.8 | 33.0±5.5 | 35.2±5.0 | **0.040** | 0.34 | 31.3 |
| Creatinine, μmol/L | 64.7±13.3 | 98.8±77.2 | 63.0±32.0 | 0.13 | 0.13 | 59 |
| Direct bilirubin, μmol/L | 3.8±1.5 | 4.4±2.0 | 4.4±3.2 | 0.41 | 0.82 | 4.9 |
| Alanine aminotransferase, U/L | 54.5±37.6 | 39.4±25.7 | 43.3±53.7 | 0.31 | 0.84 | 42 |
| Aspartate aminotransferase, U/L | 79.4±48.7 | 84.7±64.1 | 44.9±42.5 | 0.83 | 0.11 | 157 |
| Glucose^h^ | 6.9±2.2 | 9.9±2.3 | NA | 0.02 | NA | 12.0 |
| PCT, g/L | 0.29±0.27 | 0.33±0.25 | 0.75±0.73 | 0.70 | 0.11 | 0.83 |
| C-reactive protein, mg/dl | 60.5±32.3 | 43.6±27.2 | 42.1±29.4 | 0.26 | 0.91 | 35.1 |
| Erythrocyte sedimentation rate, mm/h | 56.9±26.0 | 41.4±29.3 | 48.6±36.4 | 0.31 | 0.70 | 11 |
| Lactate dehydrogenase, U/L | 489.1±243.7 | 640±260.3 | 255.8±95.2 | 0.20 | **0.0015** | 1120 |
| Creatine kinase, U/L | 559.0±610.9 | 476.0±707 | 470.5±1087.3 | 0.78 | 0.99 | 2179 |
| PaO_2_/FiO_2_, mmHg | 217.3±145.0 | 136.3±60.0 | 124.1±73.2 | 0.13 | 0.67 | 77 |
| Days for abnormal PaO_2_/FiO_2_ | 7.8±6.4 | 21.1±15.4 | 18.3±11.4 | **0.010** | 0.65 | 46(before death) |
| **Treatment strategy** |  |  |  |  |  |  |
| Oseltamivir use, days | 14.5±4.3 | 18.1±10.8 | 1.9±5.4 ^i^ | 0.28 | **0.0001** | 34 |
| Antibiotic treatment, days | 13.9±6.6 | 23.2±11.9 | 23.4±10.9 | **0.017** | 0.83 | 24 |
| Corticoid use, n (%) | 10(76.9) | 9(100) | 8(53.3) | 0.36 | **0.049** | 1 |
| Corticoid dosage,mg | 427.5±299.4 | 1014.4±646.0 | 1035.3±2163.7 | **0.009** | 0.98 | 1280 |
| Corticoid use, days | 14.1±9.6 | 23.9±9.3 | 13.6±32.1 | **0.027** | 0.36 | 25 |
| Gamma Immunoglubulin dosage,g | 127.3±65.2 | 294.4±127.3 | 56.8±113.8 | **0.007** | **0.001** | 350 |
| Oxygen requirement, n (%) | 8(61.5) | 8(88.9) | 14(93.3) | 0.353 | 0.70 | 1 |
| Oxygen support duration, days | 2.9±2.9 | 17.2±14.8 | 33.6±35.6 | **0.003** | 0.18 | 24 |
| Invasive mechanical ventilation, n (%) | 0 | 5（55.6） | 8(53.3) | **0.011** | 0.75 | 1 |
| ICU admission, n (%) | 0 | 3(33.3) | 9(60) | 0.11 | 0.40 | 1 |
| ECMO, n (%) | 0 | 0 | 0 | NA | NA | 0 |
| **Complications** |  |  |  |  |  |  |
| Acute liver damage, n (%) | 5(38.5) | 3(33.3) | 2(13.3) | 0.84 | 0.52 | 1 |
| Severe ARDS, n (%) | 8(61.5) | 8(88.9) | 1(6.7) | 0.35 | **0.0003** | 1 |
| Shock, n (%) | 0 | 1(11.1) | 0 | 0.85 | 0.79 | 1 |
| Acute renal failure, n (%) | 0 | 1(11.1) | 0 | 0.85 | 0.79 | 1 |
| Myocarditis, n (%) | 0 | 2(22.2) | 0 | 0.30 | 0.25 | 1 |
| Septic shock, n (%) | 0 | 3(33.3) | 1(6.7) | 0.11 | 0.26 | 1 |
| Multilobar infiltration, n (%) | 13(100) | 9(100) | 13(86.7) | NA | 0.70 | 1 |
| Pleural effusion, n (%) | 8(61.5) | 8(88.9) | 5(33.3) | 0.35 | **0.026** | 1 |
| Hospital stay,days(mean ± SD) | 15.5±7.1 | 30.0±11.2 | 40.0±33.2 | **0.0047** | 0.40 | 46 |
| Death, n (%) | 0 | 4(44.4) | 5(33.3) | **0.036** | 0.91 | 1 |

^a^ H7N9 patients without bacterial co-infection in the lung are shown here as the H7N9 controls.

^b^ H7N9+*A. baumannii*: H7N9 patients coinfected by Acinetobacter baumannii in the lung.

^c^ *A. baumannii*: Patients with *Acinetobacter baumannii* related pneumonia are shown here as *A. baumannii* controls..

^d^ The P value between H7N9 group and H7N9+ *A. baumannii.* The data presented as the number (percentage) of patients were analyzed by Chi-square tests, otherwise were analyzed by t test.

^e^ The P value between H7N9+*A. baumannii* group and *A. baumannii* group. The data presented as the number (percentage) of patients were analyzed by Chi-square tests, otherwise were analyzed by t test.

^f^ The case B4 was the patient whose *A. baumannii* genomes were sequenced.

^g^ Data are presented as the number (percentage) of patients unless indicated otherwise.

^h^ The glucose levels shown herein are from the patients excluding the ones with diabetes.

^i^ Only two patients took the Oseltamivir for 20 and 8 days, respectively.
