# Supplementary material for: Clinical, immunological and bacteriological characteristics of H7N9 patients nosocomially co-infected by Acinetobacter Baumannii: a case control study
Source: BMC Infect Dis. 2018 Dec 14;18:664. doi: 10.1186/s12879-018-3447-4 (PMC6295110; doi:10.1186/s12879-018-3447-4)
Supplement: Supplementary file 4 — Table S4. The multivariate analysis of the risk factors of H7N9 patients co-infected by A. baumannii (n = 13) compared to H7N9 control patients (n = 9). (DOCX 32 kb) [file 12879_2018_3447_MOESM4_ESM.docx]

**Table S4. The multivariate analysis of the risk factors of H7N9 patients co-infected by A. baumannii (n=13) compared to H7N9 control patients (n=9).**

| **Variates** | **Univariate analysis** | | | | **Multivariate analysis**^b^ | | | |
| --- | --- | --- | --- | --- | --- | --- | --- | --- |
|  | ***P* value** | **OR** | **95% CI** | | ***P* value** | **OR** | **95% CI** | |
| **Risk factors** |  |  |  |  |  |  |  |  |
| Oseltamivir | 0.282 | 1.07 | 0.946 | 1.209 |  |  |  |  |
| Antibiotics | 0.04 | 1.134 | 1.006 | 1.279 |  |  |  |  |
| Corticosteroid | 0.056 | 1.004 | 1 | 1.008 |  |  |  |  |
| gammaimmuno | 0.039 | 1.022 | 1.001 | 1.043 | 0.156 | 1.020 | 0.993 | 1.048 |
| IMV^a^ | 0.012 | 24 | 2.038 | 282.7 | 0.050 | 19.028 | 1.004 | 360.5 |
| **Clinical indicators** |  |  |  |  |  |  |  |  |
| CD3 | 0.039 | 0.99 | 0.98 | 0.999 |  |  |  |  |
| CD4 | 0.067 | 0.986 | 0.971 | 1.001 | 0.065 | 0.978 | 0.955 | 1.001 |
| CD8 | 0.076 | 0.985 | 0.969 | 1.002 |  |  |  |  |
| IL-6 | 0.157 | 2.879 | 0.665 | 12.464 |  |  |  |  |
| IL-8 | 0.103 | 2.175 | 0.856 | 5.527 |  |  |  |  |
| CRP | 0.249 | 0.981 | 0.949 | 1.014 |  |  |  |  |
| PCT | 0.679 | 2.088 | 0.064 | 68.516 |  |  |  |  |

^a^Invasive mechanical ventilation.

^b^The data for factors with P>0.2 were not shown in multivariate analysis.
