# Supplementary material for: Clinical, immunological and bacteriological characteristics of H7N9 patients nosocomially co-infected by Acinetobacter Baumannii: a case control study
Source: BMC Infect Dis. 2018 Dec 14;18:664. doi: 10.1186/s12879-018-3447-4 (PMC6295110; doi:10.1186/s12879-018-3447-4)
Supplement: Supplementary file 5 — Table S5. The multivariate analysis of the clinical risk factors and indicators of H7N9 patients co-infected by A. baumannii (n = 13) compared to the group (n = 24) combined of H7N9 control and A. baumannii control patients. (DOCX 32 kb) [file 12879_2018_3447_MOESM5_ESM.docx]

**Table S5. The multivariate analysis of the clinical risk factors and indicators of H7N9 patients co-infected by *A. baumannii* (n=13) compared to the group (n=24) combined of H7N9 control and *A. baumannii* control patients.**

| **Variates** | **Univariate analysis** | | | | **Multivariate analysis**^b^ | | | |
| --- | --- | --- | --- | --- | --- | --- | --- | --- |
|  | ***P* value** | **OR** | **95% CI** | | ***P* value** | **OR** | **95% CI** | |
| **Risk factors** |  |  |  |  |  |  |  |  |
| Oseltamivir | 0.02 | 1.131 | 1.019 | 1.256 |  |  |  |  |
| Antibiotics | 0.256 | 1.043 | 0.97 | 1.12 |  |  |  |  |
| Corticosteroid | 0.615 | 1 | 1 | 1.001 |  |  |  |  |
| gammaimmuno | 0.008 | 1.014 | 1.003 | 1.024 | 0.011 | 1.013 | 1.003 | 1.024 |
| IMV^a^ | 0.058 | 4.75 | 0.946 | 23.845 |  |  |  |  |
| **Clinical indicators** |  |  |  |  |  |  |  |  |
| CD3 | 0.02 | 0.988 | 0.979 | 0.998 |  |  |  |  |
| CD4 | 0.029 | 0.982 | 0.966 | 0.998 | 0.027 | 0.973 | 0.95 | 0.997 |
| CD8 | 0.046 | 0.987 | 0.974 | 1 |  |  |  |  |
| IL-6 | 0.157 | 2.879 | 0.665 | 12.464 |  |  |  |  |
| IL-8 | 0.103 | 2.175 | 0.856 | 5.527 |  |  |  |  |
| CRP | 0.585 | 0.992 | 0.964 | 1.021 |  |  |  |  |
| PCT | 0.348 | 0.383 | 0.051 | 2.849 |  |  |  |  |

^a^Invasive mechanical ventilation.

^b^The data for factors with P>0.2 were not shown in multivariate analysis.
