# Supplementary figures and images for: Clinical, immunological and bacteriological characteristics of H7N9 patients nosocomially co-infected by Acinetobacter Baumannii: a case control study
Source: BMC Infect Dis. 2018 Dec 14;18:664. doi: 10.1186/s12879-018-3447-4 (PMC6295110; doi:10.1186/s12879-018-3447-4)

Supplemental Figure S1

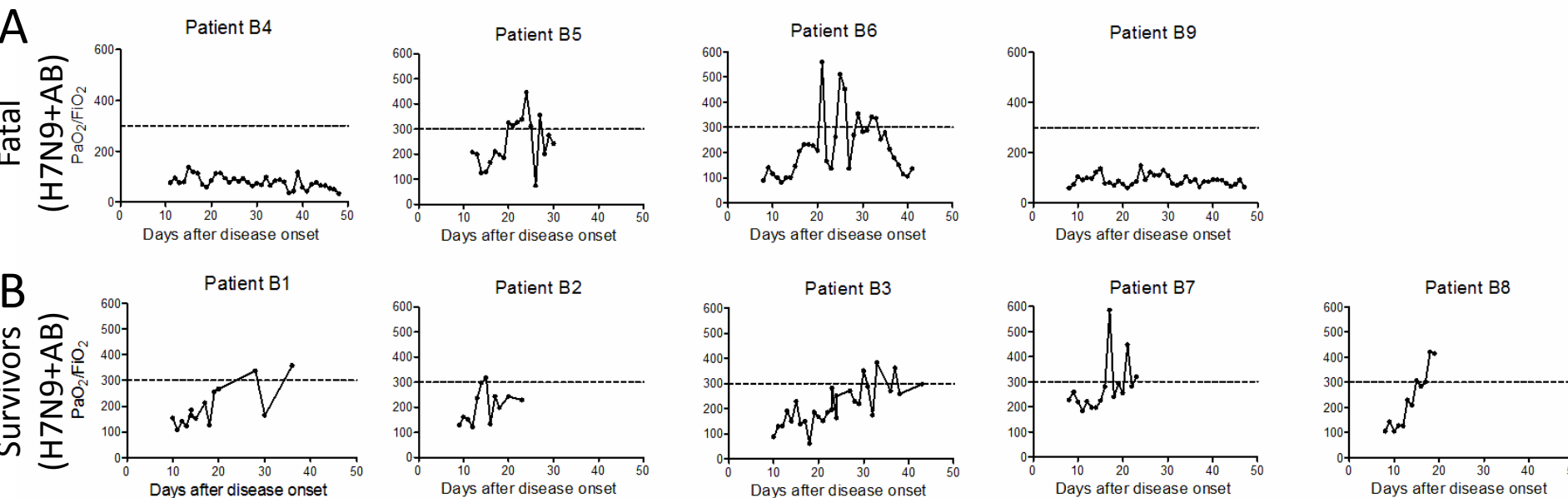

Supplement: Supplementary file 6 — Figure S1. The longitudinal variation of oxygenation index (PaO2/FiO2) in the H7N9 patients co-infected by A. baumannii. (PDF 190 kb) [file 12879_2018_3447_MOESM6_ESM.pdf]

Supplemental Figure S2

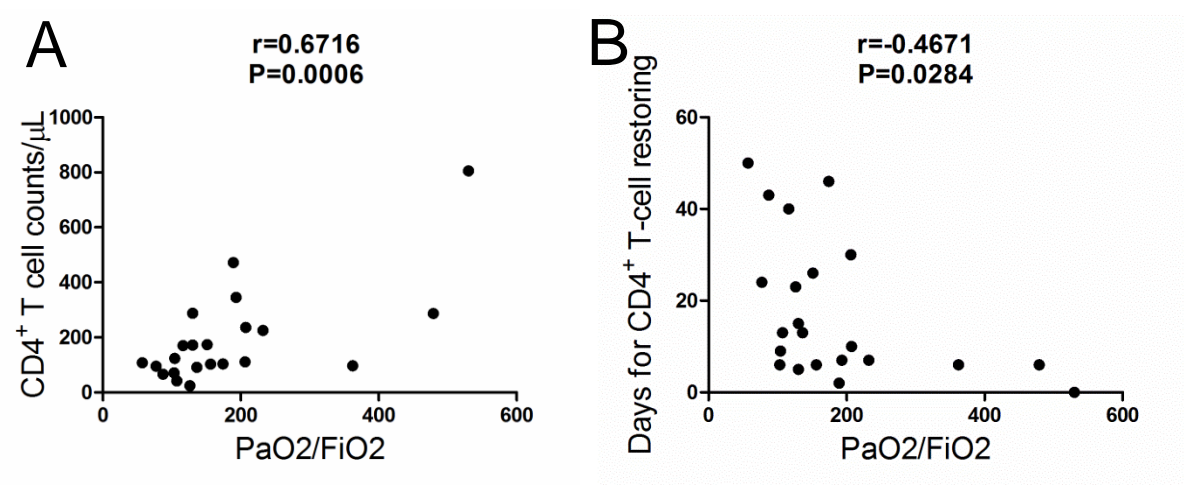

Supplement: Supplementary file 7 — Figure S2. The association of T-cell counts with oxygenation index (PaO2/FiO2) in the H7N9 patients. (PDF 237 kb) [file 12879_2018_3447_MOESM7_ESM.pdf]

Supplemental Figure S3

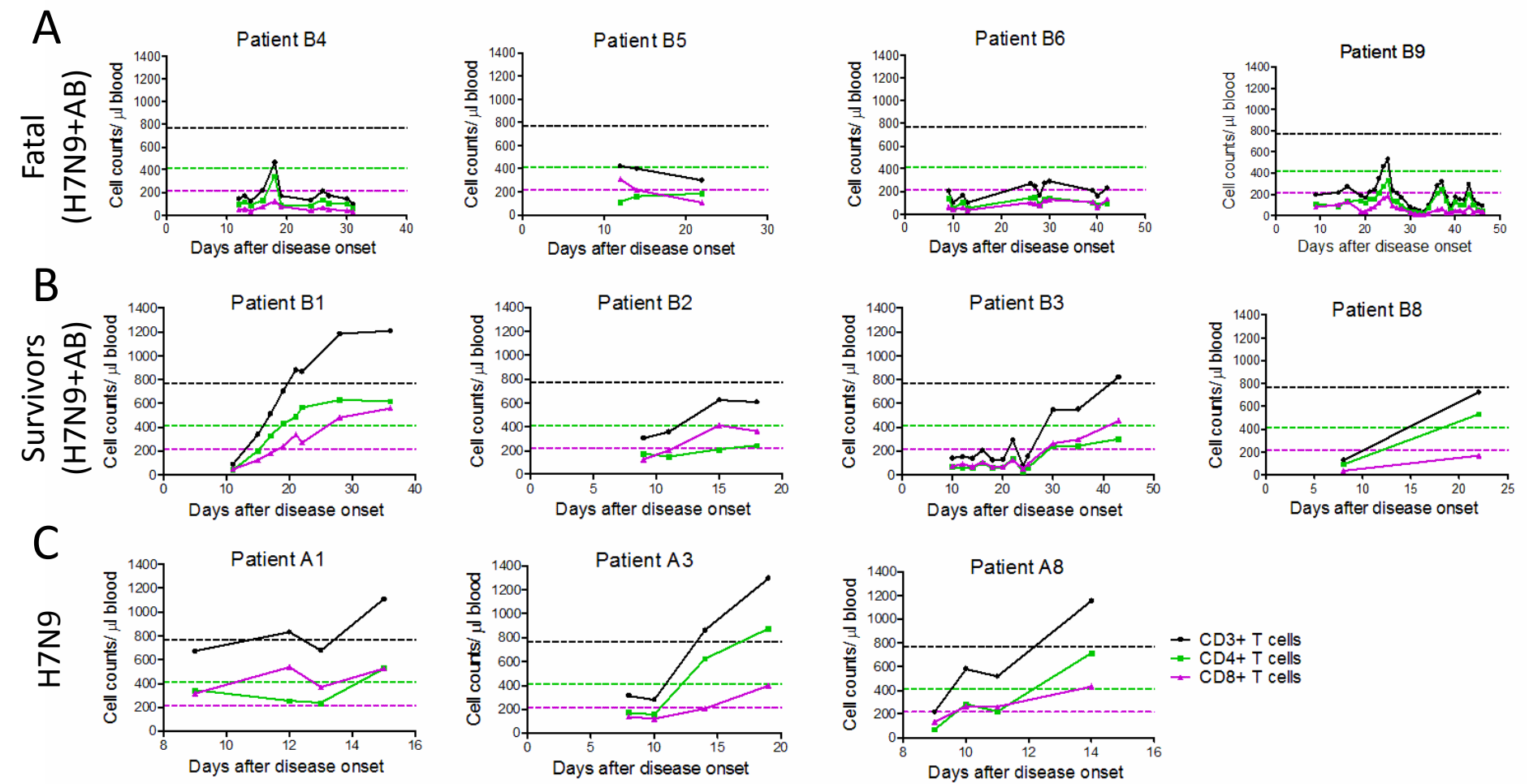

Supplement: Supplementary file 8 — Figure S3. The longitudinal trends of CD3+ T-cell counts, CD4+ T-cell counts and CD8+ T-cell counts among the H7N9 patients. (PDF 210 kb) [file 12879_2018_3447_MOESM8_ESM.pdf]

Supplemental Figure S4

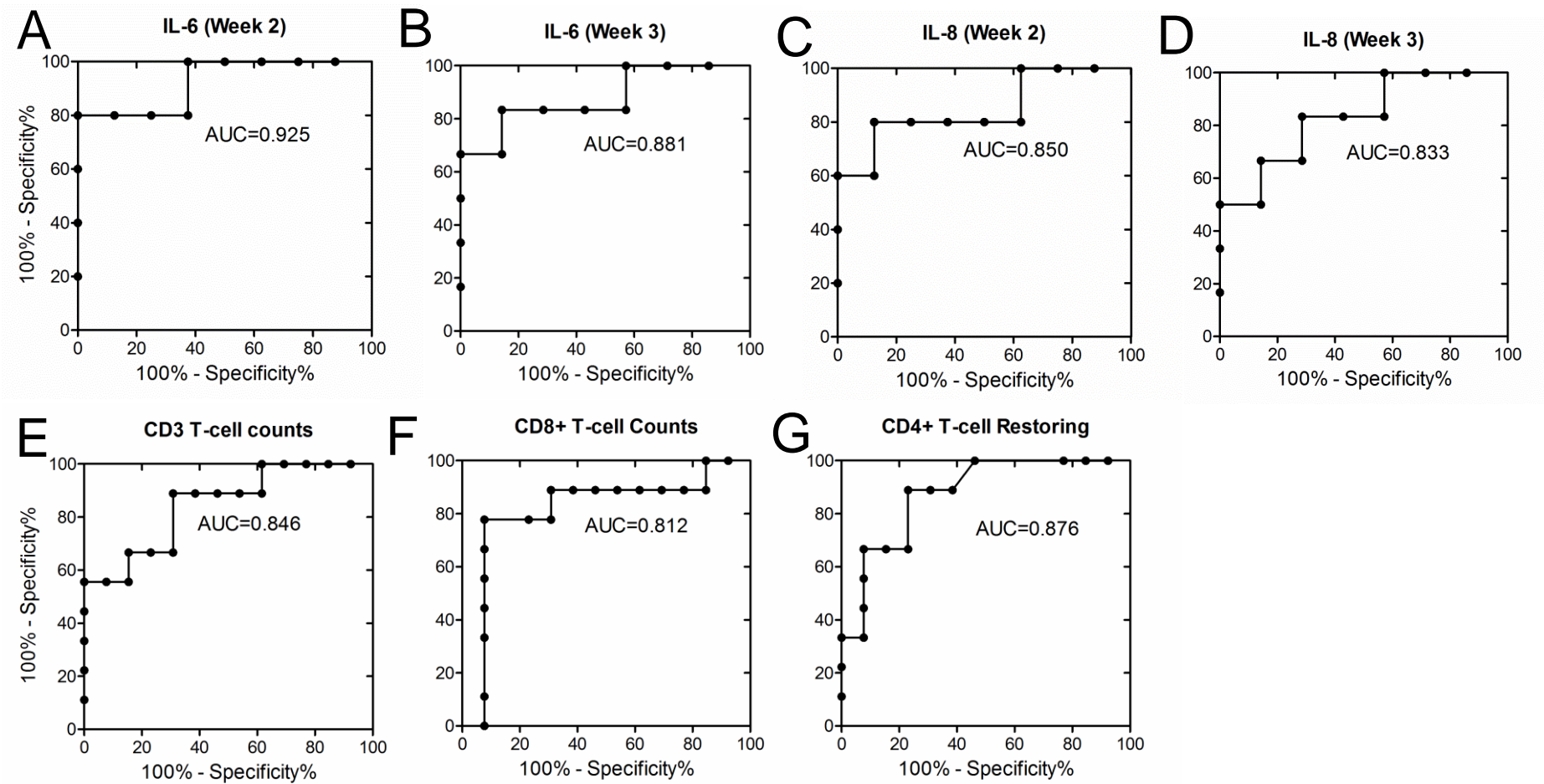

Supplement: Supplementary file 9 — Figure S4. ROC curve of the plasma levels of cytokines and T-cell characteristics in H7N9 patients. (PDF 431 kb) [file 12879_2018_3447_MOESM9_ESM.pdf]

Supplemental Figure S5

Fatal

Survivors

(H7N9+AB)

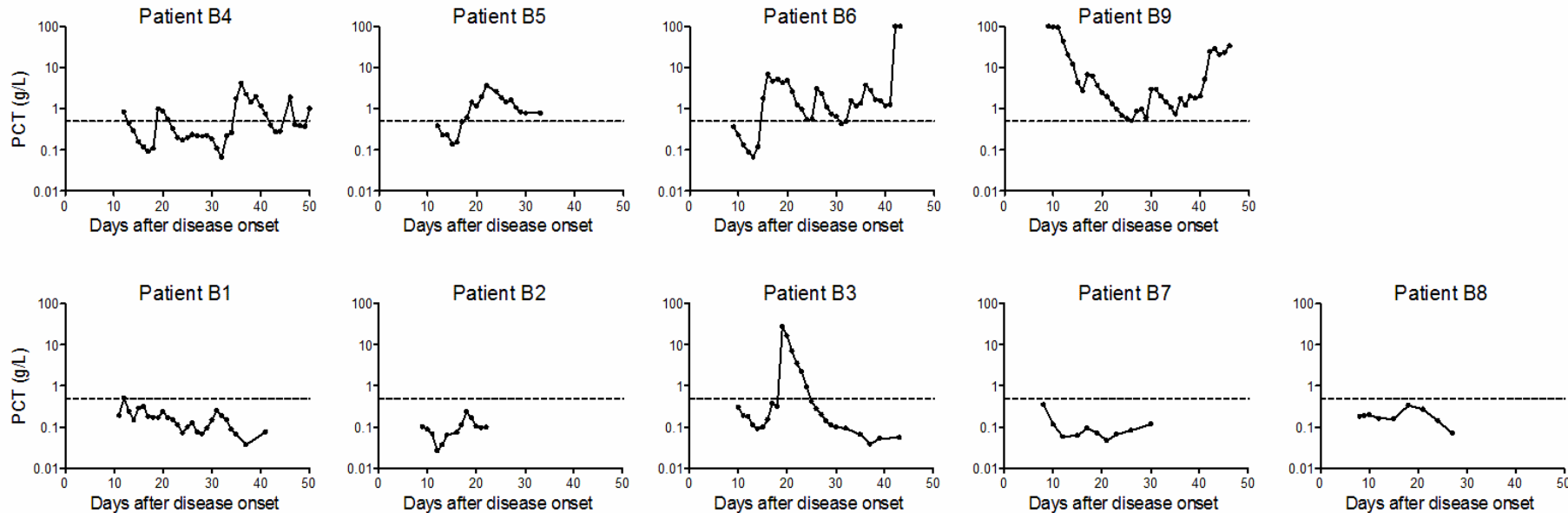

Supplement: Supplementary file 10 — Figure S5. The longitudinal variation of PCT in the H7N9 patients co-infected by A. baumannii. (PDF 185 kb) [file 12879_2018_3447_MOESM10_ESM.pdf]

Supplemental Figure S6

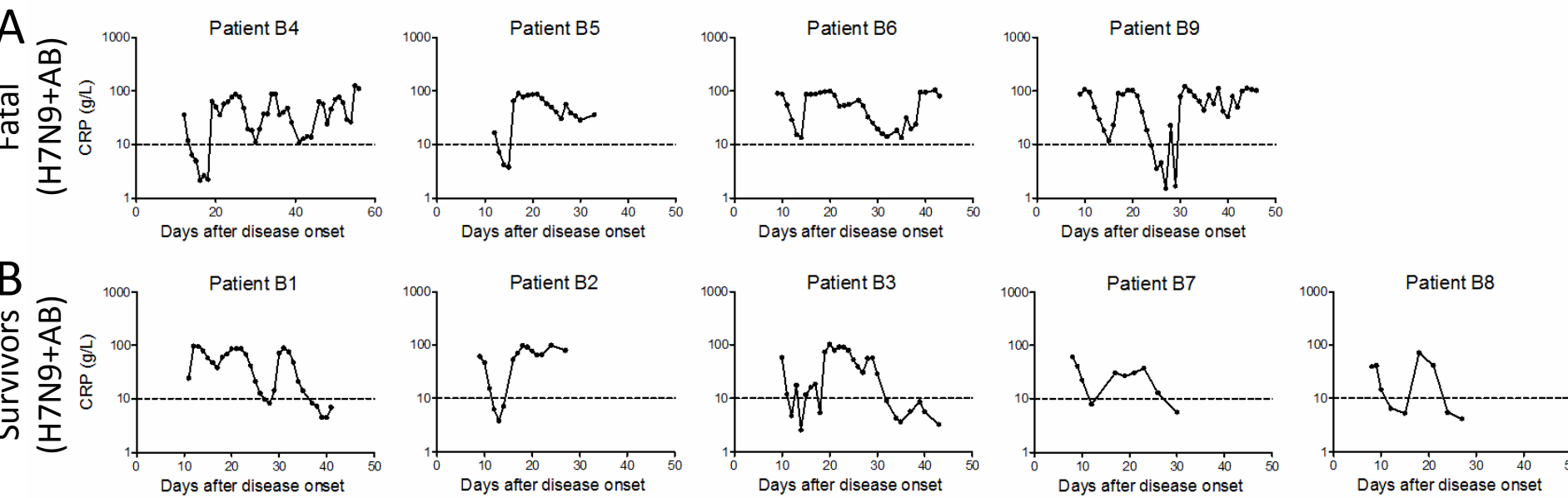

Supplement: Supplementary file 11 — Figure S6. The longitudinal variation of CRP in the H7N9 patients co-infected by A. baumannii. (PDF 188 kb) [file 12879_2018_3447_MOESM11_ESM.pdf]

## Supplemental Figure S7

A

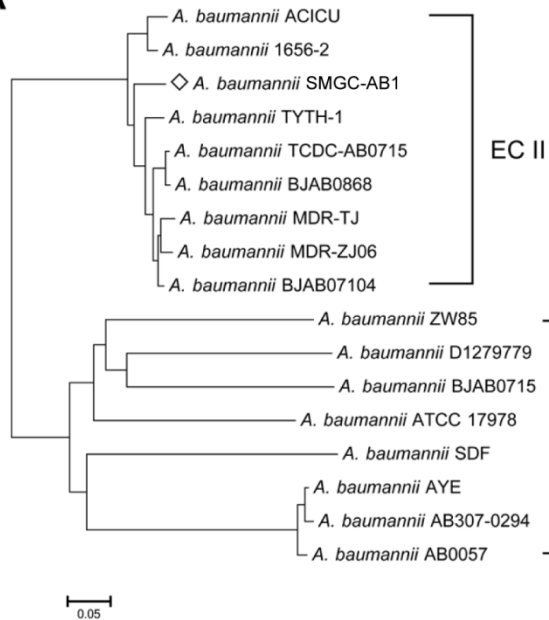

# B

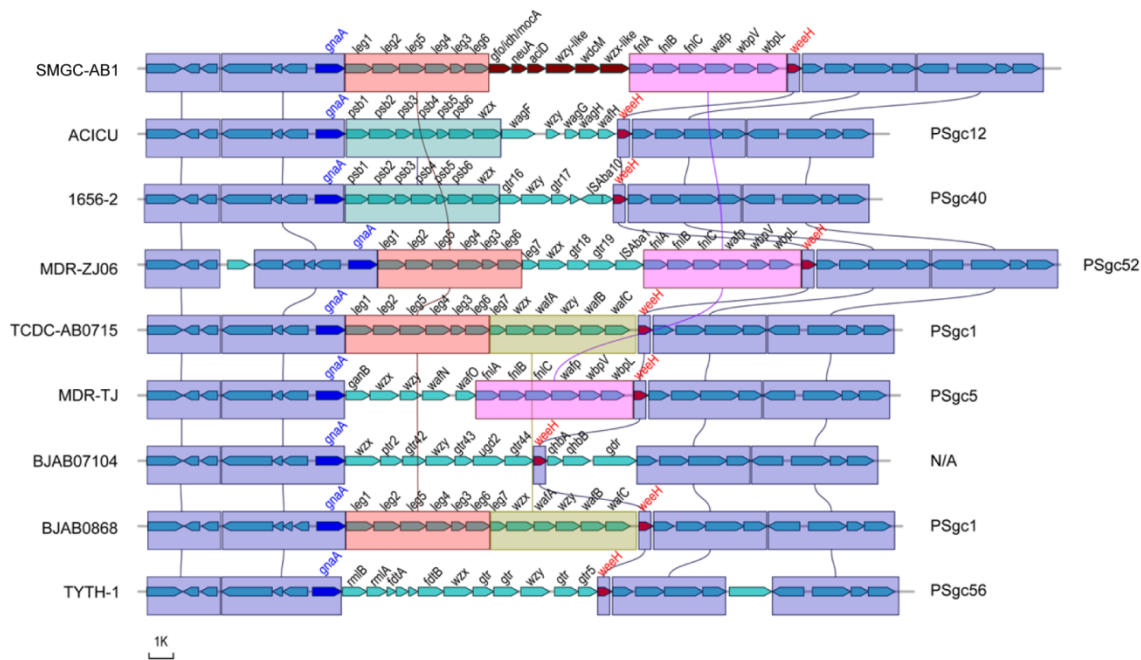

Supplement: Supplementary file 13 — Figure S7. Phylogenetic position of A. baumannii SMGC-AB1 and its polysaccharide antigen gene clusters. (PDF 624 kb) [file 12879_2018_3447_MOESM13_ESM.pdf]

Supplemental Figure S8

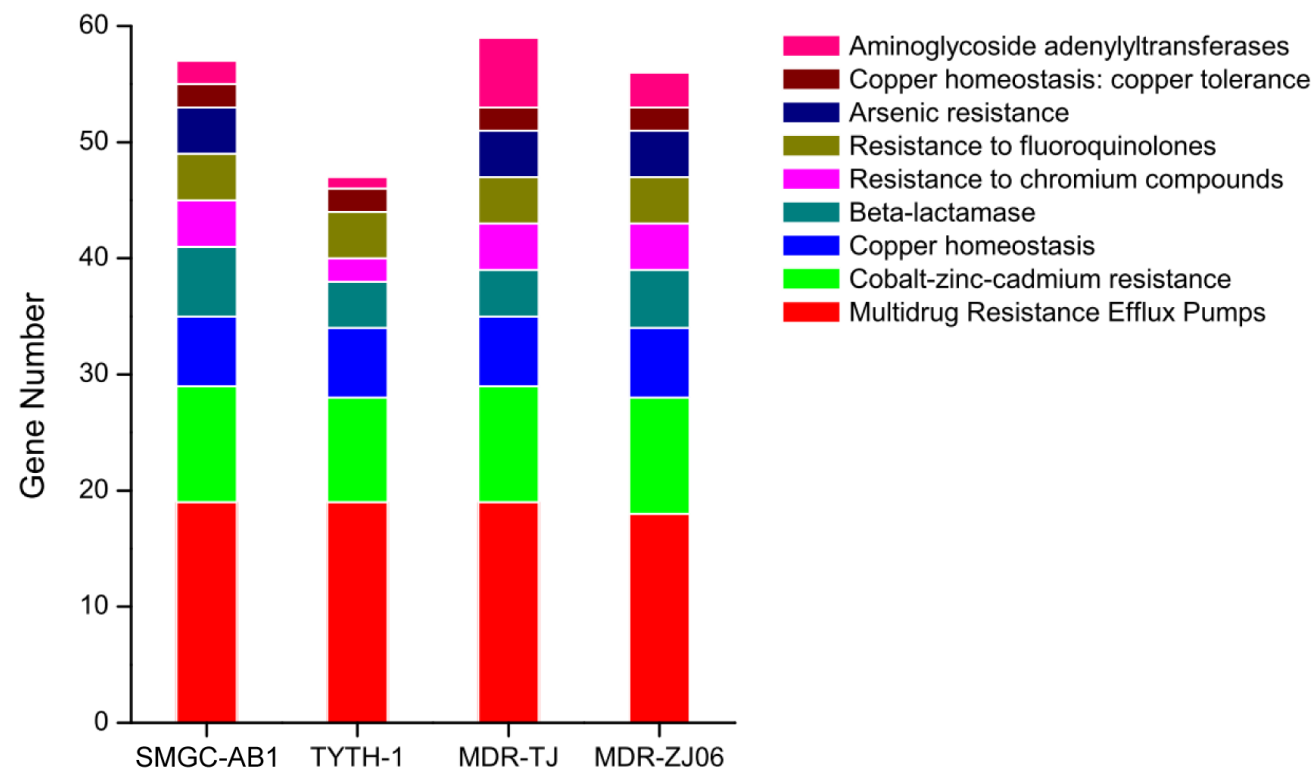

Supplement: Supplementary file 14 — Figure S8. Comparison of gene distribution in Subsystem subcategory “Resistance to antibiotics and toxic compounds”. (PDF 337 kb) [file 12879_2018_3447_MOESM14_ESM.pdf]
