# Supplementary material for: Clinical, immunological and bacteriological characteristics of H7N9 patients nosocomially co-infected by Acinetobacter Baumannii: a case control study
Source: BMC Infect Dis. 2018 Dec 14;18:664. doi: 10.1186/s12879-018-3447-4 (PMC6295110; doi:10.1186/s12879-018-3447-4)
Supplement: Supplementary file 12 — Table S6. Antibiotic susceptibility profiles for A. baumannii SMGC-AB1 and SMGC-AB2. (DOCX 32 kb) [file 12879_2018_3447_MOESM12_ESM.docx]

**Table S6.** **Antibiotic susceptibility profiles for** ***A. baumannii* SMGC-AB1 and SMGC-AB2.**

| Class | Antibiotic | **SMGC-AB1** | | **SMGC-AB2** | |
| --- | --- | --- | --- | --- | --- |
|  |  | MIC (μg/ml) | R/I/S | MIC (μg/ml) | R/I/S |
| Aminoglycosides | Amikacin | >32 | R | >32 | R |
|  | Gentamicin | >8 | R | >8 | R |
| Penicillins | Ampicillin | >16 | R | >16 | R |
|  | Ampicillin/ Sulbactam | >16/8 | R | >16/8 | R |
|  | Amoxicillin/Clavulanic acid | >16/8 | R | >16/8 | R |
|  | Piperacillin | >64 | R | >64 | R |
|  | Piperacillin/Tazobactam | >64/4 | S | >64/4 | R |
| Cephalosporins | Cefotaxime | >32 | R | >32 | R |
|  | Cefazolin | >16 | R | >16 | R |
|  | Ceftazidime | >16 | R | >16 | R |
|  | Cefepime | >16 | R | >16 | R |
| Monobactam | Aztreonam | >16 | R | >16 | R |
| Carbapenems | Imipenem | >8 | R | >8 | R |
|  | Meropenem | >8 | R | >8 | R |
| Sulfonamides | Sulfamethoxazole | >2/38 | R | >2/38 | R |
| Polymyxins | Polymyxin | **≤0.5** | **S** | **>2** | **R** |
| Fluoroquinolones | Levofloxacin | >8 | R | >8 | R |
|  | Moxifloxacin | >4 | R | >4 | R |
|  | Ciprofloxacin | >2 | R | >2 | R |
| Chloramphenicol | Chloramphenicol | >16 | I | >16 | I |
| Tetracycline | Tetracycline | >8 | R | >8 | R |

R, resistant; I, intermediate; S, susceptible.
